# Depletion of Highly Abundant Protein Species from Biosamples by the Use of a Branched Silicon Nanopillar On-Chip Platform

**DOI:** 10.1021/acs.analchem.1c03506

**Published:** 2021-10-20

**Authors:** Ella Borberg, Sofiya Pashko, Vlad Koren, Larisa Burstein, Fernando Patolsky

**Affiliations:** † School of Chemistry, Faculty of Exact Sciences, Tel Aviv University, Tel Aviv 69978, Israel; ‡ George S. Wise Faculty of Life Sciences, Tel Aviv University, Tel Aviv 69978, Israel; § The Wolfson Applied Materials Research Centre, Tel Aviv University, Tel Aviv 69978, Israel; ∥ Department of Materials Science and Engineering, the Iby and Aladar Fleischman Faculty of Engineering, Tel Aviv University, Tel Aviv 69978, Israel

## Abstract

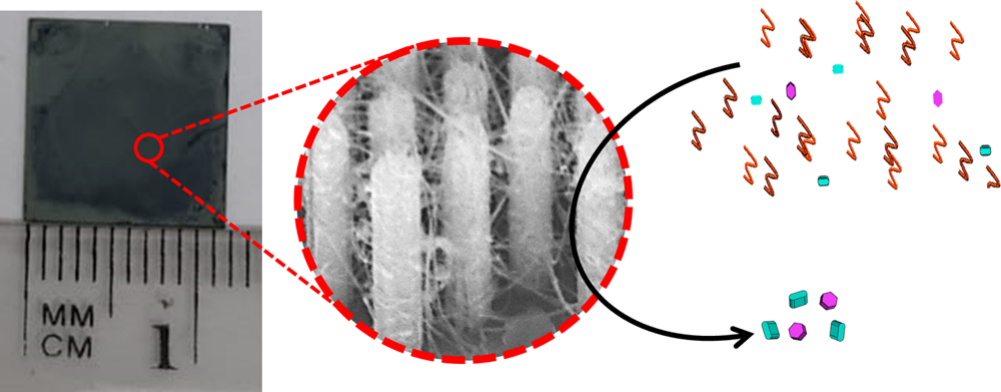

Highly abundant serum proteins tend to mask the low- and ultralow-abundance proteins, making low-abundance species detection extremely challenging. While traditional highly abundant protein depletion techniques are effective, they suffer from nonspecific binding problems and laborious sample manipulation procedures, and the kinetics of release of current separation systems is inadequately long, causing dilution of the eluted low-abundance protein samples. Here, we introduce an on-chip light-controlled reusable platform for the direct and fast depletion of highly abundant proteins from serum biosamples. Our nanoarrays display fast and highly selective depletion capabilities, up to 99% depletion of highly abundant protein species, with no undesired depletion effects on the concentration of low-abundance protein biomarkers. Displaying an ultrahigh surface area, ∼3400 m^2^ g^–1^, alongside a light-triggerable ultrafast release, this platform allows for a high depletion performance, together with high-yield reusability capabilities. Furthermore, this nanostructured light-controlled separation device could easily be integrated with downstream analytical technologies in a single lab-on-a-chip platform.

## Introduction

A current focus within proteomics is to identify serum protein biomarkers that correlate to disease and the disease stage and may be targeted for drug therapy or may reflect a change in the physiological status in response to therapeutic intervention.^1,2^ Developments in proteomic profiling techniques have increased sensitivity and throughput, yet capturing the dynamic state of an entire proteome, such as the serum proteome, still facing multiple challenges, one of the greatest being the separation and detection of target low-abundance proteins from complex biosamples.^3−6^ Blood samples typically contain more than 10,000 different proteins in a concentration range varying over 10 orders of magnitude.^7^ The sensing of new protein biomarkers, usually present at very low concentrations, is hindered by the “masking” effect of highly abundant proteins.^8,9^ For instance, the 22 most abundant proteins represent approximately 99% of the bulk mass of the total protein content in human plasma, probably leaving hundreds of thousands of other proteins in the rest of ca. 1% of the plasma protein mass.^10^ Most abundant serum proteins include human serum albumin (HSA), IgGs, IgAs, haptoglobin, α-1-*anti*-trypsin, and transferrin and make up to 85% of the total serum protein mass.^11^ These proteins mask the low- and ultralow-abundance proteins, making the low-abundance species detection extremely challenging, since their concentrations are lower than the detection limit of most analytical instruments.^12^ Mass spectrometry analysis is one of the most sensitive analytical tools currently available for proteomics; however, the dynamic range of detection limits it. Therefore, effective depletion of these highly abundant proteins during the biosample preparation process has become largely widespread, to provide higher sensitivity and achieve a broader proteome coverage, principally of the low-abundance protein species in the concentration range of ng/mL and lower.

HSA is the most abundant protein in serum, present at a concentration of 39–51 mg/mL.^13^ Traditional HSA depletion techniques implement the hydrophobic dye Cibacron Blue, which has a high affinity for albumin.^14−18^ While it is of relatively low cost^19−21^ and able to remove the majority of HSA, this technique additionally removes lower abundant protein biomarkers from serum samples.^22^ Other popular methods include centrifugal ultrafiltration^9,23^ and immunoaffinity devices.^24−26^ Centrifugal ultrafiltration is limited in reproducibility, as the size of membrane pores varies, with normally distributed smaller and larger pores.^9^ Immunoaffinity devices have been shown, by comparative studies, to result in more controlled specific depletion than dye ligand-based depletion methods,^22,27−30^ leading to the increased use of immunoaffinity devices, made up of matrices with covalently attached antibodies against the specific highly abundant proteins. However, the effectiveness of several commercially available immunoaffinity devices has been compared by several studies, showing that these devices are vulnerable to nonspecific-binding artifacts to the device matrix.^22,31−33^ These antibody-based depletion strategies have demonstrated high efficiencies in removing the targeted high-abundance proteins. However, issues still arise concerning their reproducibility and selectivity.^10,22,28,31,34,35^

Particularly, there are two main open questions: Are these systems capable of removing the specific target proteins reproducibly? Is there any considerable loss of nontarget low-abundance proteins during the depletion of the high-abundance proteins, probably through nonselective binding mechanisms? Preferably, potential losses of nontarget low-abundance proteins must be minimized during removal of multiple high-abundance proteins, but in such cases, where losses do exist, they should be reproducible if the depletion strategy is to be used for quantitative biomarker discovery studies.

Additionally, the kinetics of elution of current separation systems is inadequately long, requiring the use of elution solutions, potentially causing denaturation of both the immunoassay platform and the adsorbed and nonadsorbed target proteins, resulting in the undesired dilution of the protein samples. Furthermore, these methods lack compatibility to integration on a single platform with downstream technologies, such as sensing devices,^36−42^ high-throughput systems, lab-on-chip microfluidic devices, and complementary metal oxide semiconductor fabrication routes.

Nanomaterials and specifically nanowires^43−47^ have been shown to be versatile excellent candidates for the fabrication of devices in a broad range of applications such as electronics,^48^ optics,^49^ biosciences,^50^ medical diagnosis,^51^ and energy storage.^52^ In particular, previous studies from our group^53,54^ presented the potential of silicon nanopillar (SiNP)^55−69^ arrays as an on-chip reusable light-controlled nanostructured platform for the selective and quantitative separation, desalting, preconcentration, and direct analysis of complex biosamples. Analytes were separated selectively from raw biosamples using antibody–photoacid-modified SiNP arrays of ultrahigh surface area and high binding affinity and specificity, followed by the light-controlled rapid release of the tightly bound target molecules to controlled liquid medium, within only 1 min.

This SiNP-based platform, although shown to be highly effective in the separation of low-abundance proteins,^53^ will still require a dramatic increase in the active capturing surface area to be suitable to perform full depletion of high-abundance proteins in the concentration range of tens–hundreds of mg/mL. Thus, growing dense silicon nanowire (SiNW) branches on the SiNP elements, to form a branched-SiNP (BSiNPs) array, may dramatically increase the platform’s binding surface capacity, thus meeting the aforementioned needed requirements. Importantly, the previous studies demonstrated the selectivity of the SiNP arrays for the capture of proteins of interest, along with the absence of nonspecific binding of unwanted protein species, even for the ultralow-abundance species. Additionally, the kinetics of release of current separation systems is inadequately long and results in the undesired dilution of the eluted protein samples. Thus, we here make use of a photoacid-modified surface that greatly accelerates the release of captured protein species and allows for a reusable capture-and-release proteomic platform. HSA release is triggered by light activating a covalently bonded photoacid molecular agent, causing a drastic pH change near the SiNP surfaces, thus leading to the pH-caused dissociation of antibody–antigen pairs. Photoacids are aromatic organic molecules that exhibit high acidity in their first excited electronic state, greater by many orders of magnitude than their weak acid properties in their ground electronic state. These materials have been widely studied in several applications,^70−74^ including the light-triggered “pH drop” caused by photoactivation of photoacid molecules applied in SiNW-based field-effect transistor devices for the on-surface modulation of protein affinity to an antibody.^75^ Photoactivation of photoacids creates a pH change that is triggerable and reversible and does not involve buffer-handling limitations. In addition, the modulation of photoacid molecules’ surface density, along with the applied light intensity, allows achieving a controlled “surface pH” and the subsequent rapid seconds-long release of the tightly captured protein species from the inter-SiNP cavity into the eluted bulk solution.

Here, we demonstrate the development of a BSiNP-based on-chip light-controlled reusable nanostructured depletion platform for multiple abundant proteins, directly out from serum biosamples. We demonstrate the fast, ca. 20 min, and selective serum albumin (SA) and additional abundant protein depletion capabilities of our BSiNP arrays, modified with a specific antibody and a photoacid molecule, displaying an ultrahigh surface area, alongside a triggerable ultrafast elution into selected medium for further downstream analysis. Furthermore, this BSiNP light-controlled separation device could be easily integrated with downstream analytical technologies in a single platform and successfully applied for the multiplex, real-time, and ultrasensitive detection of protein biomarkers.

## Experimental Section

### Materials and Chemicals

Acetone (9005-68, J. T. Baker), isopropanol (IPA; 9079-05, J. T. Baker), deionized water (DIW; 18 MΩ·cm), phosphate buffer (PB; 10 mM, pH 8.5), glutaraldehyde solution (50 wt % in H_2_O, G7651, Sigma-Aldrich), (3-aminopropyl)-dimethyl-ethoxysilane (APDMES, SIA0603.0-5g, Gelest), enhanced green fluorescent protein (eGFP; JM-4999-100, MBL), eGFP antibody (GTX33910, GeneTex), silicon substrate wafer (polished, P-type, ⟨100⟩, 1–10 Ω·cm, 380 ± 15 μm thickness, WaferPro), toluene (244511, Sigma-Aldrich), phosphate-buffered saline (PBS; P4417, Sigma-Aldrich), bovine SA (BSA) (Abcam, ab64009), BSA antibody (Abcam, ab2460), protein A/G (21186, Thermo Fisher), CA-15.3 antibody (CA1531-M, Alfa Diagnostic), gold etchant (GE-8148, Transene), and polystyrene bead suspension (10%, 0.5 μm, Sigma).

### Fabrication of the SiNP Array Using Metal-Assisted Etching

As previously described,^54^ the steps utilized in the fabrication of the SiNP array are as follows: 1% polystyrene bead suspension solution is prepared by centrifuging 100 μL of 10% polystyrene bead suspension at 4500 rpm for 16 min, separating the polystyrene beads from the water, and 1 mL of 3% Tween 80 in methanol was added. The suspension is dispersed with shaking. Next, a silicon substrate is washed with acetone, IPA, and DIW and then dried with N_2_. Polystyrene beads are spread by spin coating 18 μL of suspension solution at 475 rpm for 1 min (for 4 cm^2^ wafer). Polystyrene beads are then minimized to 250 nm diameter using plasma etching (50 sccm O_2_, 40 mTorr, 30 W, 6 min), and the surface is coated in a Ag film by E-beam deposition (45 nm, 1 Å/s). SiNP formation is achieved with silicon wet etching in a solution of 4.6 M HF and 0.44 M H_2_O_2_ for 9 min. Silver residues are removed with HNO_3_, and polystyrene residues are removed with O_2_ plasma (10 min, 100 W, 0.200 Torr).

### Fabrication of the BSiNP Array

The SiNP array is first washed with acetone and IPA, dried with N_2_, and cleaned in O_2_ plasma for 10 min (100 W, 0.200 Torr). Gold nanoparticles are formed on the SiNPs’ surface in an electroless deposition process by soaking the array for 30 min in 20% DIW, 80% ethanol, 0.05 M HF, 110 μM NaAuCl_4_, and 1% Tween 80. SiNWs are synthesized in a chemical vapor deposition (CVD) system by a 2 min’ process using 20 sccm argon and 5 sccm SiH_4_ at a pressure of 25 Torr and 460 °C. Finally, gold residues are removed with a gold etchant solution, and the array is washed with DIW and dried with N_2_.

### Brunauer–Emmett–Teller Measurement Information

Particle Testing Authority performed measurements via Dr. Golik Scientific Solutions, utilizing a TriStar II Plus instrument. Analysis adsorptive: Kr, analysis bath temperature: 77.300 K, thermal correction: no, equilibration interval: 10 s, low-pressure dose: none, sample density: 1.000 g/cm^3^, automatic degas: no, correlation coefficient: 0.9999157, molecular cross-sectional area: 0.2100 nm^2^ sample was kept under vacuum at 100 °C for 960 min, analyzed per ISO 9277; the reference material(s) used for instrument performance verification is available upon request.

### Preparation of 8-Acetoxy-pyrene-1,3,6-trisulfonyl Chloride out of 1-Hydroxypyrene-1,3,6-trisulfonate

8-Acetoxy-pyrene-1,3,6-trisulfonyl chloride out of 1-hydroxypyrene-1,3,6-trisulfonate (HPTS) preparation is as previously described:^75^

A total of 20 g of trisodium-1-hydroxypyrene-1,3,6-trisulfonate was dissolved in 30 mL of NaOH (2 M) and cooled to about 0 °C. A total of 5 g of acetic anhydride (4.8 mL) was added dropwise and stirred for 2 h. A total of 20 mL of ethanol is added; the precipitate is then filtrated, washed three times with 10 mL of ethanol, and dried under reduced pressure for 24 h.

A total of 5 g of precipitate are placed with 150 mL of toluene in a round-bottomed flask, equipped with an automatic water separator (Dean–Stark trap) and a condenser, and refluxed for 2 h. The mixture is then cool to about 60 °C; 6 mL of oxalyl chloride and two drops of dimethylformamide are added and refluxed for 8 h. The precipitate is removed by filtration, and the solvent is removed under reduced pressure. The solid residue is dried under a vacuum for 24 h.

### Antibody and HPTS Immobilization on the SiNP Array

The SiNP array is first washed with acetone and IPA, dried with N_2_, and cleaned in O_2_ plasma for 10 min (100 W, 0.200 Torr). The array is then soaked in 100% APDMES for 3 h under an argon environment. The array is washed with IPA and dried in an oven for 30 min at 115 °C. The SiNP array was modified with an 8-acetoxy-pyrene-1,3,6-trisulfonyl chloride layer with 12 h of incubation with 8-acetoxy-pyrene-1,3,6-trisulfonyl chloride and pyridine and then exposure of the phenol functional group with a saturated sodium bicarbonate solution. Next, the array is soaked in 8.3% glutaraldehyde containing 12 mM sodium cyanoborohydride for 3 h and washed with DIW, acetone, IPA, and DIW again.

Antibody modification is achieved by incubating an array in antibody solution at 10–600 μg/mL in PB containing 12 mM sodium cyanoborohydride, at 4 °C, overnight, and blocking open aldehyde groups is done by soaking the array in 100 mM ethanolamine solution containing 12 mM sodium cyanoborohydride in PB (pH 8.5), for 3 h under shaking at about 30 rpm; finally, the array is washed in PB.

### X-ray Photoelectron Spectroscopy Measurement Information

X-ray photoelectron spectroscopy (XPS) measurements were performed in UHV (2.5 × 10^–10^ Torr base pressure) using 5600 a multi-technique system (PHI, USA). The samples were irradiated with an Al Kα monochromated source (1486.6 eV), and the outcome electrons were analyzed using a spherical capacitor analyzer using the slit aperture of 0.8 mm. In the case of samples’ charging during the measurements, a charge neutralizer and additional mathematical peak shifting were used, with C 1s at 285 eV taken as an energy reference. The samples were analyzed on their surfaces only.

### Adsorption–Desorption Experimental Protocol

The protocol is portrayed in Supporting Information Figure S1.

First, a 1.2 × 1.2 cm^2^ immunomodified array is washed in PB; then, the array is placed on a clean hydrophobic surface. A total of 230–600 μL of a sample (serum or spiked solution) is pipetted on top of the array surface. After stirring, 5 μL of the sample is periodically taken out and optically measured for specific species concentrations at 0, 2, 4, 8, 16, 20, 40, and 120 min. A total of 190–560 μL of the residual sample is pipetted off the arrays’ surface; the array is washed gently in PBS and placed on a clean hydrophobic surface. A total of 500 μL of PB (or any required medium) is pipetted on top of the arrays’ surface. A light source (400 nm, 50 mW/cm^2^) is turned on. After stirring, 5 μL of the sample is periodically taken out and optically measured for specific species concentrations at 0, 2, 4, 8, 16, 20, 40, and 120 min. A total of 460 μL of the residual medium is pipetted off the arrays’ surface, and the array is washed in PB and left soaked in PB at 4 °C for storage.

### Optical Measurement Information

A series of fluorescence/absorbance measurements were performed using a commercial fluorescence scanner (Tecan Infinite M200), using corning black 384 plates (3820). Fluorescence calibration curves are shown in Supporting Information Figure S2.

### Experimental Repetition and Error Bars

All adsorption and desorption experiments shown in Figures 3 and 4 were conducted at least five times (*n* ≥ 5). Error bars were chosen as the highest variation measured for the experiment type.

## Results

### BSiNP Array Fabrication and Chemical Modification

**Figure 1:**
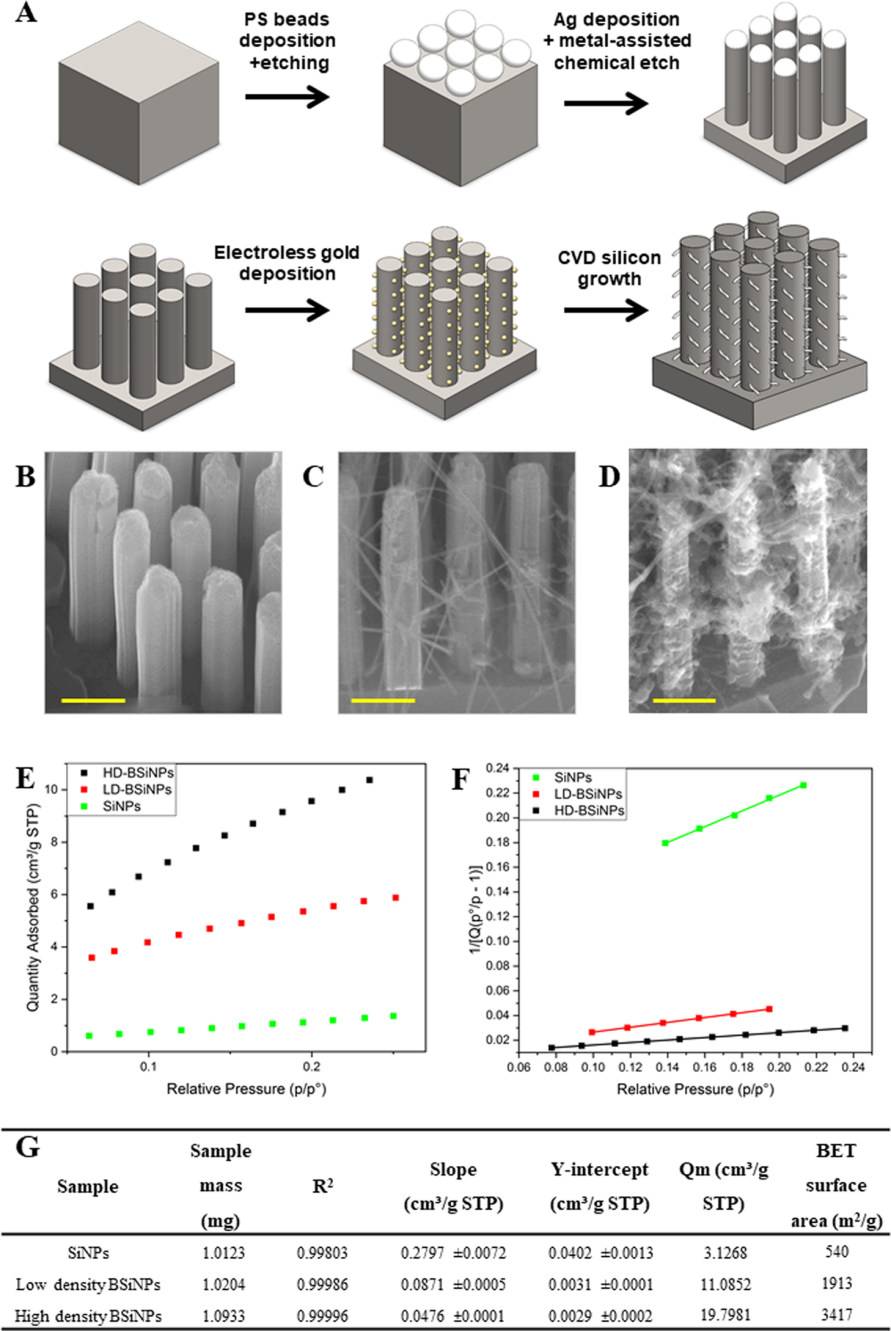
BSiNP array fabrication and surface area characterization. (A) Fabrication process of the BSiNP array with silicon metal-assisted wet etching, followed by CVD-based growth of SiNBs. SEM images of a representative SiNP array, (B) low-density BSiNPs, (C) and high-density BSiNPs, (D). All images were taken in the cross-sectional view at 20° angle. Scale bars: 0.5 μm. (E) BET isotherms of Kr adsorption fitted in linear plots, 77.30 K. (F) Measured BET Kr adsorption surface area plots, 77.30 K. (G) Summarized results of BET surface area analysis.

The fabrication process is schematically depicted in Figure 1A. First, a monolayer of polystyrene beads^76^ was used as an etching mask for a metal-assisted wet etching step with a HF/H_2_O_2_ mixture as an etchant and oxidant.^77^ A deposited silver metal film was used as a catalyst, forming a vertical SiNP array, of 3–20 μm SiNP height (see the Experimental Section).

To further increase the surface area, silicon nanobranches (SiNBs) were grown on the surface of SiNPs to form BSiNP arrays. Shortly, electroless deposition of gold on the surface of the nanopillars was achieved by soaking SiNP arrays in AuCl_4_/HF solution, followed by CVD using the gold nanoparticles as catalysts for the growth of SiNWs via the vapor–liquid–solid mechanism.^78^ The SiNB growth process used SiH_4_ as a reactant. The rate of the SiNW growth was ∼1 μm/min.

Scanning electron microscopy (SEM) images of the different resulting arrays, Figure 1B–D, show the difference in SiNB density that can be reached by adjustment of the time of electroless deposition and CVD growth process.

Surface areas of the resulting arrays measured by the Brunauer–Emmett–Teller (BET) method using Kr gas are shown in Figure 1E–G. The adsorption isotherms for the different arrays are shown in Figure 1E. These three arrays allow a comparison of the effect of increasing branch density. Increasing branch density corresponds to rougher structures of higher porosity, and Figure 1E shows that as expected, the adsorption saturation capacities increase with increasing branch density. The data points from Figure 1E are linearly plotted in Figure 1F according to the BET adsorption isotherm equation
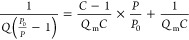
1where *P* is the partial vapor pressure of adsorbate gas in equilibrium with the surface, *P*_0_ is the saturated pressure of adsorbate gas, *Q* is the volume of gas adsorbed at standard temperature and pressure (STP), *Q*_m_ is the volume of gas adsorbed at STP to produce an apparent monolayer on the sample surface, and *C* is a dimensionless constant that is related to the enthalpy of adsorption of the adsorbate gas on the sample.

The linear parameters are summarized in Figure 1G; the SiNP array surface area reaches up to ca. 540 m^2^ g^–1^. This correlates to an increase in the geometrical surface area from a planar substrate of 1 into 500 cm^2^ after the etching of a SiNP array, comprising SiNPs of 5 μm height, 250 nm diameter, and 250 inter-NP distance. This represents a dramatic increase of more than a 500-fold active surface area in comparison to a planar device of an identical geometrical area.

A further increase in the surface area has been confirmed by BET measurements, with BSiNP arrays reaching ca. 3400 m^2^ g^–1^. Fabricating higher SiNP arrays displaying increased roughness and a more densely packed growth of Si nanobranches, by minor changes to the SiNP fabrication, gold deposition, and/or CVD process, would result in even higher increases in the surface’s active capturing area.

**Figure 2:**
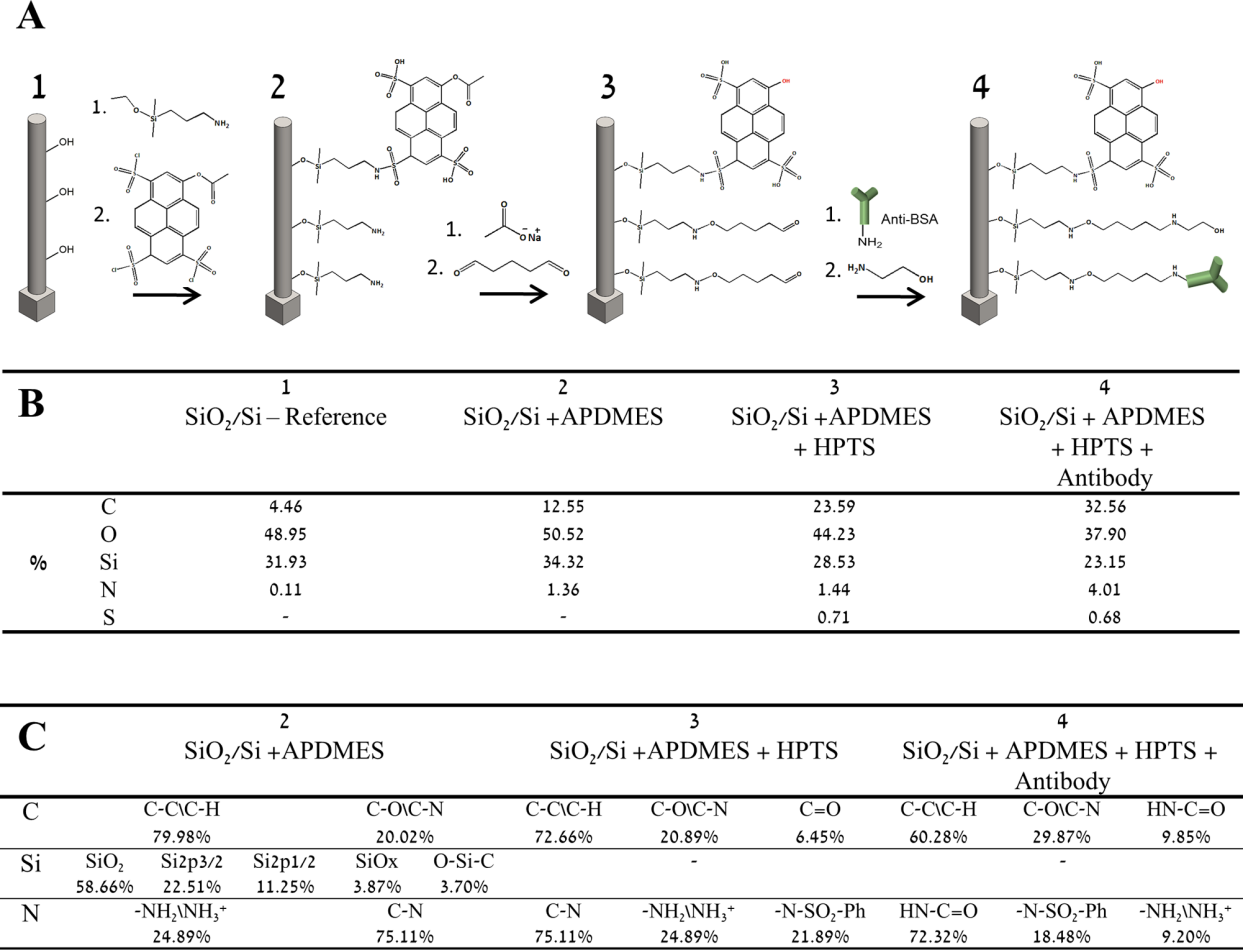
Chemical surface modification process. (A) Schematics of the chemical immobilization procedure of HPTS and antibody molecules onto the SiNP array surface. (B) X-ray photoelectron spectroscopy-analyzed atomic concentration percentages during each step of the HPTS and antibody immobilization process on the SiNP array surface. (C) Corresponding chemical bond population percentages at each modification step.

Next, BSiNP array surfaces are chemically modified, as outlined in Figure 2A, with APDMES, followed by immobilizing a derivative of 8-hydroxypyrene-1,3,6-trisulfonic acid (HPTS), 8-acetoxy-pyrene-1,3,6-trisulfonyl chloride.^53,73^ Frequently applied as a light-triggered source of protons in various studies,^53,72,73,75,79−81^ HPTS has a p*K*_a_ of ∼7.3 at the ground state and is exceptionally more acidic when photoexcited, with p*K*_a_ as low as ∼0.4. Previous fluorescence experiments verified that the photoactivated pH decrease is confined to the surface. Upon activation, surface pH was measured to be ∼3.3–3.5, while bulk pH remained unchanged at ∼7.5.^75^

Next, arrays are chemically modified with a layer of HSA-specific IgG monoclonal antibodies (additional antibodies against other abundant proteins were applied as well by chemical modification of the capturing arrays with several specific antibodies against multiple abundant targets). The modification of the BSiNP array with HPTS was verified by the use of fluorescence microscopy measurements and by XPS. Atomic concentrations are summarized in Figure 2B, and high-resolution chemical bond populations are shown in Figure 2C. APDMES immobilization onto the surface is verified by the rise in nitrogen atomic concentration, and the O–Si–C bond determined the surface coverage to be ∼3.0 × 10^13^ molecules/cm^2^ APDMES. The addition of sulfur and the N–SO_2_–Ph bond determine an HPTS surface coverage of ∼8.6 × 10^12^ molecules/cm^2^; thus, only 5% of the surface amino groups are linked to a photoacid molecule, leaving a large number of free amino groups available for further chemical immobilization of capturing antibody molecules. An IgG density of ∼1.6 × 10^13^ molecules/cm^2^ is determined by higher atomic concentrations of carbon and nitrogen and the amide bond HN–C=O.

By applying BSiNP arrays of high branch density, a maximal antibody density can be theoretically expected (60% surface coverage of 8 nm footprint IgG molecules) at 1.3 × 10^16^ molecules/cm^2^ (1 cm^2^ geometric flat area converts into ∼3000 cm^2^). Higher IgG densities (per cm^2^ geometrical area) could be reached using higher and rougher SiNP arrays or more densely grown Si nanobranches.

### Albumin Depletion Efficiency from Serum Biosamples

**Figure 3:**
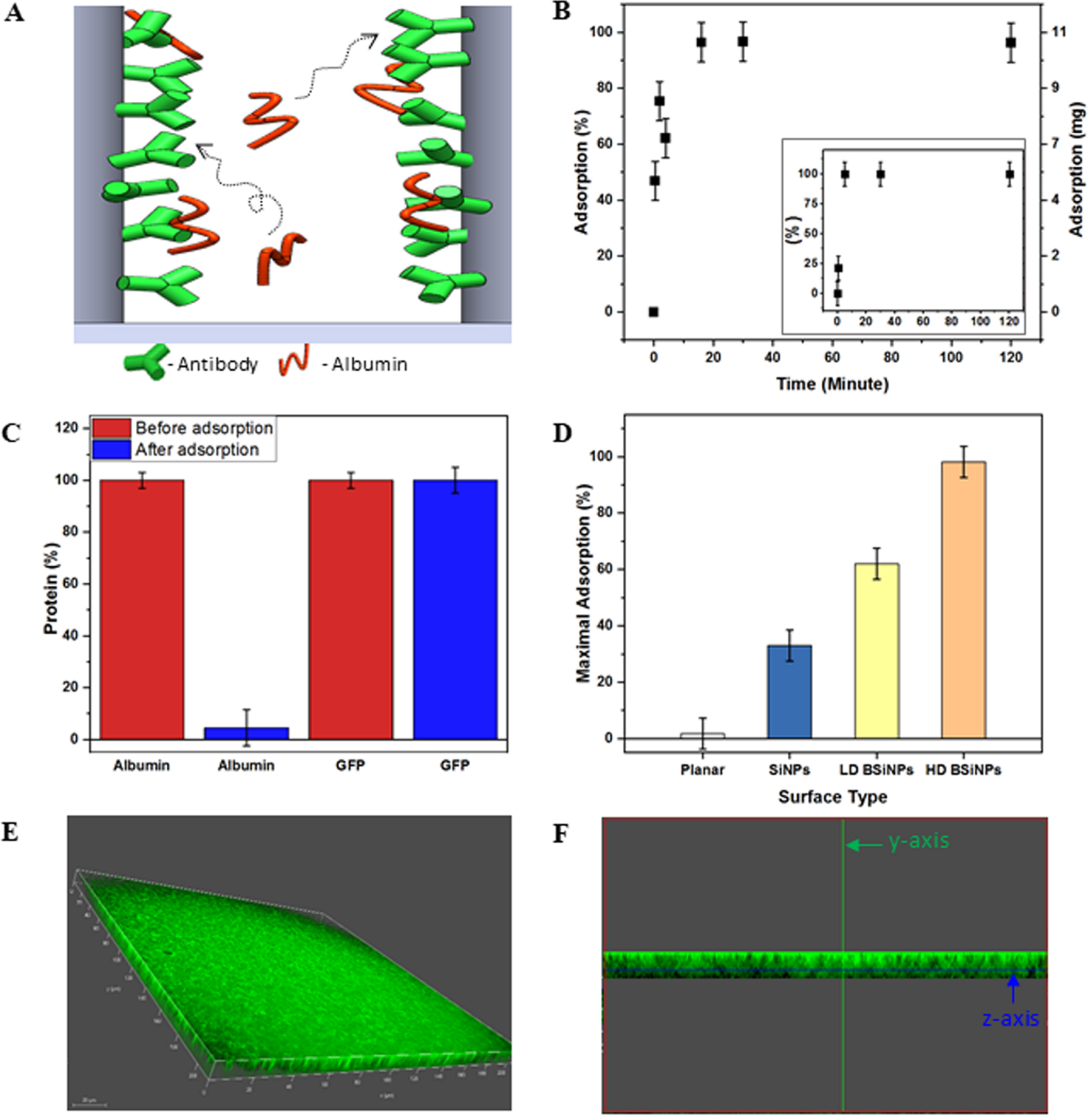
BSA depletion capabilities of the BSiNP array. (A) Schematic illustration of the albumin-trapping phenomena exhibited by the BSiNP array. (B) BSA capturing out of a 50 mg/mL BSA solution in PBS at different time points. Inset: IgG capturing out of a 3.5 mg/mL cancer antigen-15.3 solution in PBS. (C) BSA and GFP, before and after 2 h of capturing out of a 50 mg/mL BSA and 9 μg/mL GFP serum sample. (D) Maximal BSA capturing onto different arrays, from a 50 mg/mL BSA serum sample. (E) Fluorescence microscopy 3D-reconstructed image of GFP penetration into the inter-nanopillar cavities of a high-density BSiNP array and top view of the BSiNPs at 40°. (F) Cross-sectional view of the *x*-axis from the 3D-reconstructed image in (E).

Our hypothesis is that biomolecule adsorption is enhanced since molecules experience limited diffusion inside nanocavities of the nanostructured SiNP arrays, causing them to delay while being adsorbed to and desorbed from, capturing antibody molecules into the confined interpillar space repeatedly, until ultimately being released to the above bulk medium. This eventually leads to extremely prolonged effective dissociation rates, allowing our highly receptive SiNP arrays to effectively and very efficiently capture biomolecules from bulk solutions, >99%, further preventing their release back to the bulk solution based on this novel nanoconfinement-related capturing mechanism. Figure 3A schematically illustrates this concept. BSA and cancer-antigen 15.3 antibody (anti-CA15.3) protein markers have been used as a model for highly abundant protein species (50 mg/mL BSA and 3.6 mg/mL anti-CA15.3). Additionally, green fluorescent protein (GFP, 9 μg/mL) has been chosen as a model for a low-abundance protein. BSA and GFP concentrations were optically quantified using fluorescence emission at 333 and 515 nm, respectively. CA15.3-antibody has been quantified using absorption at 280 nm. BSA and GFP surface adsorption was determined by eq 2
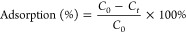
2

Figure 3B shows a BSA-capturing capacity reaching ca. 11 mg/cm^2^ within only 20 min of incubation, ca. 99% of albumin found in the used sample (220 μL of 50 mg/mL BSA solution per cm^2^ of BSiNP array). These BSA-capturing results correlate to an adsorption capacity of 478 mg/g (BSA per 10 μm × 1 cm^2^ Si wafer base for a BSiNP array). When accounting for the particularly fast capturing time reported, ∼20 min, the capturing abilities of the BSiNP array are noteworthy. BSA-capturing attempts showed to be extremely efficient, Figure 3B, with full depletion (>99%) of BSA from biosamples occurring very rapidly, achieving a plateau after only ca. 20 min of incubation. This is also the case for highly abundant IgG species depletion, seen in the inset of Figure 3B. Importantly, while albumin concentrations have diminished greatly, GFP protein concentrations in the treated biosamples are shown to be stagnant, thus demonstrating that the undesirable nonspecific adsorption of the low-abundance GFP protein is negligible, Figure 3C. This demonstrates the highly specific capturing capabilities of our BSiNP arrays. These effective capturing results are observed as well by multi-antibody-modified BSiNP arrays (four antibody species against IgGs, IgMs, HSA, and haptoglobin). Also, the capturing platform demonstrates a level of specificity highly required for the postdepletion analysis of low-abundance protein biomarkers.

As mentioned previously, a larger surface area and therefore higher capturing capacity could be easily achieved with mild alterations of the array fabrication process. This is shown in Figure 3D, where a planar substrate reaches maximal adsorption of only 4% of the total sample BSA after 2 h, capturing performance orders of magnitude lower than that demonstrated for the novel chemically modified high-density BSiNP arrays.

The depth penetrability of proteins into the nanometric interpillar cavities has been measured by 3D-reconstructed imaging after the binding of the fluorescent GFP protein, on an anti-GFP-modified high-density BSiNP array in PBS spiked with 0.3 μM GFP, as seen in Figure 3E,F. The GFP protein could be homogeneously observed from the base of the interpillar cavities through the top of the array, attesting that the protein permeability is unharmed by the high-density branches of the array.

### Light-Triggered BSA Rapid Release and Platform Reusability

Irradiation at a wavelength of 400 nm brings surface-confined HPTS molecules to their excited state, where they display a dramatic change in their p*K*_a_, from 7.4 to ∼0.4, becoming superacid molecules that rapidly expel their phenolic protons to the surrounding media. The light-triggered pH drop caused by photoactivation of the covalently bound photoacid molecules may be applied for the on-surface activation of pH-dependent chemical and biological processes, such as the dissociation of antigen–antibody pairs.^53,73,75^ The pH change has been shown in the past to depend on the surface density of the photoacid and the intensity of the light source.^72,75^ The photoacids’ surface density is controlled by the concentration of the photoacid concentration in the modification solution and the time of array incubation.^53,75^ This photoacid surface density is of critical importance and must be carefully controlled for the successful and effective light-activated release of surface-bound biomolecules.^53^

**Figure 4:**
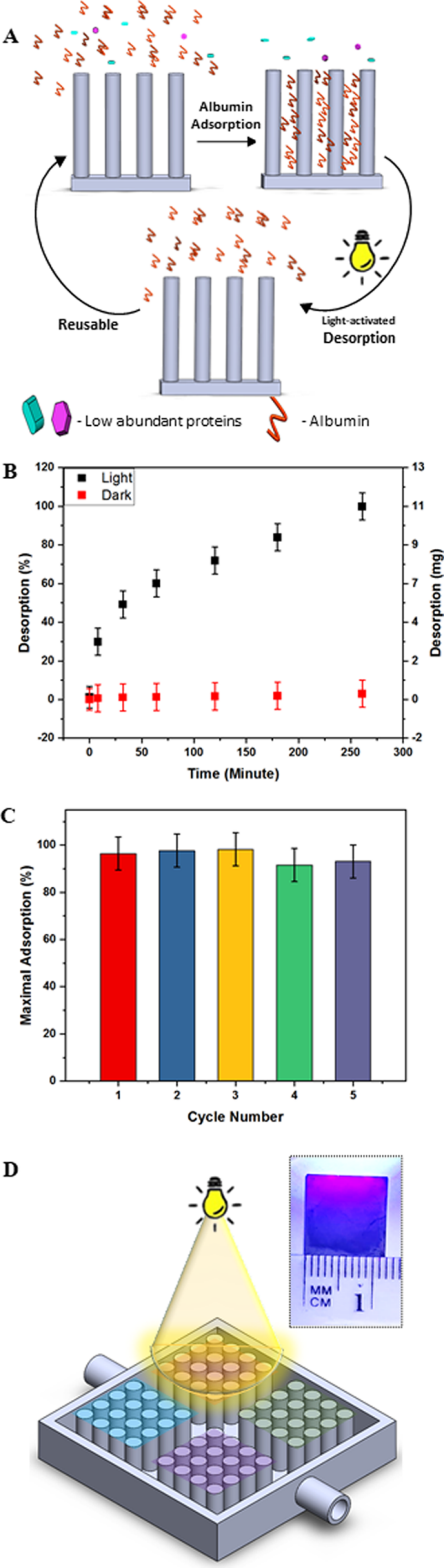
Light-activated BSA dissociation from the BSiNP array. (A) Schematics of the BSA capture–dissociation cycles. (B) BSA dissociation at different time points, under dark and light conditions. (C) Maximal BSA capture/depletion performance, from a 50 mg/mL BSA sample, at different multiple cycles of use. (D) Schematic demonstration of the SiNP platform divided into four sections of different modifications; inset: the BSiNP chip partially illuminated.

This pH-drop effect leads to fast protein desorption from the array without damaging the chemical surface modification, allowing simple reusability of the immuno-nanoarray. This is illustrated schematically in Figure 4A, showing the fast surface-bound protein molecules being released by this light-activated mechanism.

As expected, the fast and quantitative albumin capturing is followed by a highly prolonged release under dark conditions. Only up to ca. <2% of the BSA captured molecules desorb spontaneously from BSiNP arrays after long periods of >2 h, Figure 4B, red curve, meaning that the BSA effective dissociation rate is *k*_off_ ≈ 1.2 × 10^–4^%/min. Therefore, the full release of surface-captured BSA molecules would be achieved after ca. 830,000 min (about 578 days). As previously discussed,^53^ we believe that the molecules bound at the top of the BSiNP array represent the relatively faster ca. <2% of the observed spontaneous release.

Notably and in contrast to results under dark conditions, activation of the surface-confined HPTS molecules by light irradiation (400 nm, 50 mW/cm^2^) allows for the immediate rapid dissociation of the captured BSA species, with *k*_off_ ≈ 0.5%/min, 5 orders of magnitude faster than the *k*_off_ measured under “dark” conditions. Previous experiments demonstrated that the pH drop is experienced in close vicinity to the SiNP surface, controlled by light intensity and photoacid surface concentration, and is responsible for the experimentally observed rapid release of surface-captured biomolecules.^73^

Furthermore, while the biosample fractions containing the most abundant proteins were previously presumed to be diagnostically unimportant and usually left unanalyzed, the light-activated desorption process allows for their analysis and more importantly for platform reusability. Remarkably, no decrease in the capture-and-release effectiveness of the BSiNP arrays was observed after performing five consecutive cycles of protein capture and light-triggered release, Figure 4C. We, therefore, infer that the platform could be effectively used for multiple capture-and-release cycles.

As schematically clarified in Figure 4D, in the future, our platform could be utilized in both albumin and immunoglobulin depletion, with one array divided into several sections modified with different specific binding agents in each section. This, followed by illumination of a required section, could lead to depletion of abundant species from biosamples and allow for retrieval of each species separately for downstream analysis.

## Conclusions

The development of an on-chip light-controlled reusable nanostructured depletion platform of abundant protein species directly out from serum samples has been presented. We demonstrated fast, complete, and selective SA depletion capabilities (and multiple-abundant protein depletion in a single chip), up to >99% in ca. 20 min, brought by strongly trapping the highly abundant proteins inside the NP cavities chemically modified with specific antibodies, with no apparent detrimental effects on the concentrations of low-abundance protein biomarkers. Increasing the surface area of our nanostructured platform, from ∼540 to ∼3400 m^2^ g^–1^, showed to triple the platform’s protein-capturing capability. An ultrahigh surface area, alongside a light-triggerable ultrafast release, allows for rapid depletion performance, together with platform reusability. Furthermore, this BSiNP light-controlled separation device could easily be integrated with downstream analytical technologies in a single lab-on-a-chip platform, for fast and quantitative depletion of highly abundant proteins from a broad variety of liquid biosamples such as blood, serum, interstitial fluid, and saliva.^5,38,53,54,82^

## Supporting Information Available

Adsorption–desorption experimental protocol, fluorescence calibration curves of albumin, and 3D fluorescence microscopy results (PDF)
