# Supplementary material for: Depletion of Highly Abundant Protein Species from Biosamples by the Use of a Branched Silicon Nanopillar On-Chip Platform
Source: Anal Chem. 2021 Oct 20;93(43):14527–36. doi: 10.1021/acs.analchem.1c03506 (PMC8592501; doi:10.1021/acs.analchem.1c03506)
Supplement: Supplementary file 1 — ac1c03506_si_001.pdf [file ac1c03506_si_001.pdf]

Supporting information

Depletion of Highly Abundant Protein Species from Biosamples by the Use of a Branched Silicon Nano-Pillars On-Chip Platform

Ella Borberg[1], Sofiya Pashko[2], Vlad Koren[1], Larisa Burstein[3], and Fernando Patolsky[1,4*].

1. School of Chemistry, Faculty of Exact Sciences, Tel Aviv University, Tel Aviv, 69978, Israel.
2. George S. Wise Faculty of Life Sciences, Tel Aviv University, Tel Aviv, 69978, Israel.
3. The Wolfson Applied Materials Research Centre, Tel-Aviv University, Tel-Aviv 69978, Israel.
4. Department of Materials Science and Engineering, the Iby and Aladar Fleischman Faculty of Engineering, Tel Aviv University, Tel Aviv 69978, Israel.

Email: fernando@post.tau.ac.il

Table of Contents

Figure S1 3

Figure S2 4

Figure S3 4

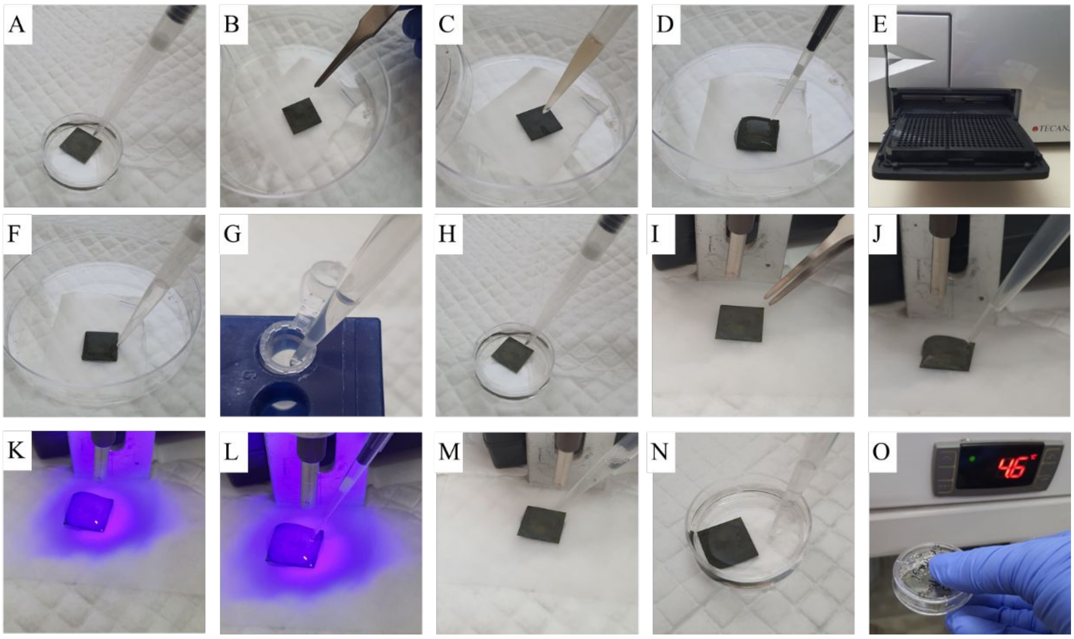

Figure S1

Capturing-release experimental protocol portrayal. **A,** 1.2x1.2 cm^2 immuno-modified array is washed in PB. **B,** The array is placed on a clean hydrophobic surface. **C,** 230-600 µl of a sample are pipetted on top of the array surface. After stirring, 5 µl of the sample are periodically taken out, **D,** and optically measured for specific species concentrations, **E,** at 0, 2, 4, 8, 16, 20, 40, and 120 minutes. **F,** 190-560 µl of the residual sample are pipetted off the arrays' surface and kept if needed**, G**. **H,** the array is washed gently in PBS. **I,** the array is placed on a clean hydrophobic surface under a light source. **J,** 500 µl of PB (or any required medium) are pipetted on top of the arrays' surface. **K,** the light source (400 nm, 50 mW/cm2) is turned on. After stirring, 5 µl of the sample are periodically taken out, **L,** and optically measured for specific species concentrations at 0, 2, 4, 8, 16, 20, 40, and 120 minutes. **M,** 460 µl of the residual medium are pipetted off the arrays' surface. **N,** the array is washed in PB. **O,** the array is left soaked in PB at 4 ^{0}C for storage. Notably, the whole process can be simply automated by the use of a fluidic cell.

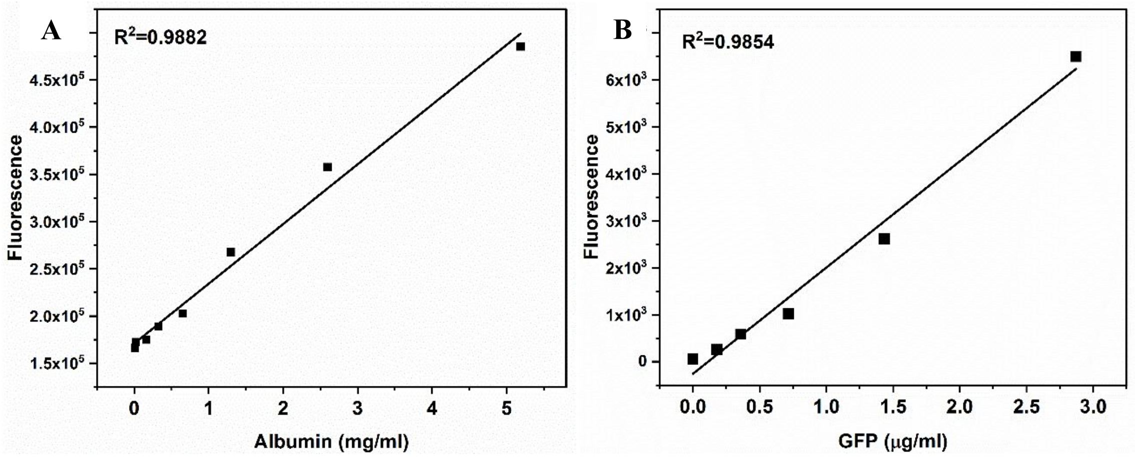

Figure S2

Fluorescence calibration curves of albumin, **A,** and GFP, **B,** in PB (black curves), and PBS (blue curves).

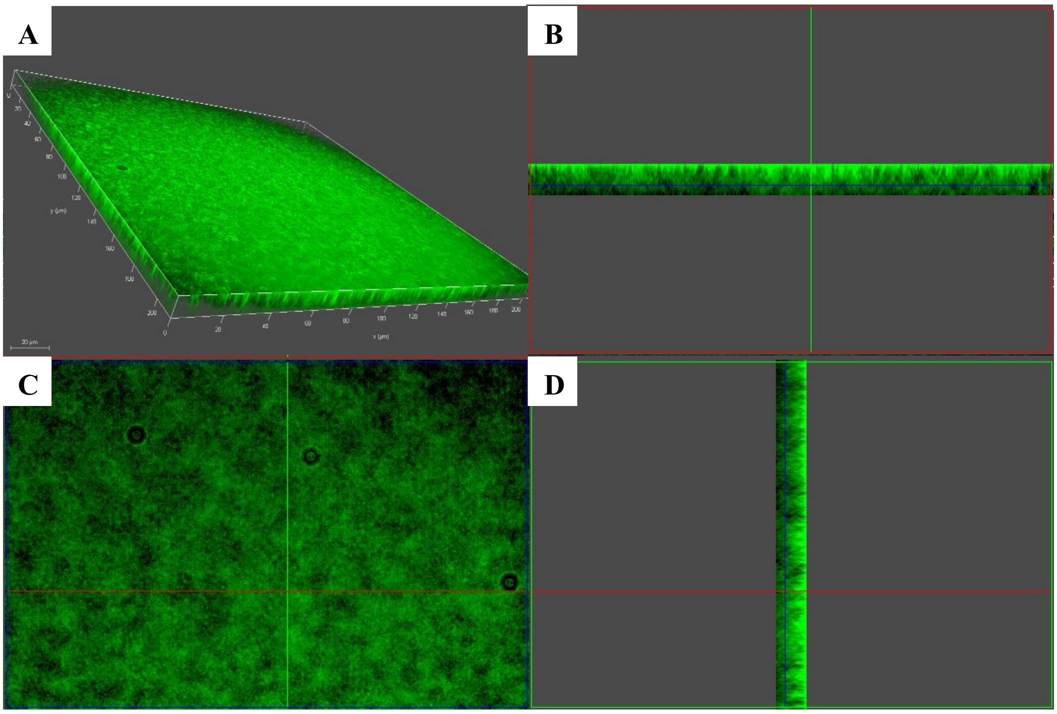

Figure S3

Fluorescence microscopy 3D-reconstructed images of GFP penetration into the inter-nanopillar cavities of a high-density BSiNPs array. **A,** Top-view of the BSiNPs at 40 degrees tilting angle. **B,** Cross-section view of the x-axis. **C,** Bottom section of the BSiNPs inter-nanopillar cavities. **D,** Lateral cross-section view of the nanopillars arrays
